# Body Mass Index and Mortality in Adults Hospitalized with COVID-19 at a Tertiary Referral Hospital in Northern Jordan, 2020–2021: A Single-Center Retrospective Secondary Analysis

**DOI:** 10.3390/healthcare14213739

**Published:** 2026-11-03

**Authors:** Alhareth Ahmad, Osama Abo Alrob, Mohammad B. Nusair, Sayer Al-Azzam, Reema Karasneh

**Affiliations:** 1 Pharmacological and Diagnostic Research Center, Faculty of Pharmacy, Al-Ahliyya Amman University, Amman 19328, Jordan; 2 Department of Basic Medical Sciences, Faculty of Medicine, Yarmouk University, Irbid 21163, Jordan; osama.yousef@yu.edu.jo; 3 Department of Clinical Pharmacy and Pharmacy Practice, Faculty of Pharmacy, Yarmouk University, Irbid 21163, Jordan; nusair@yu.edu.jo; 4 Department of Clinical Pharmacy, Faculty of Pharmacy, Jordan University of Science and Technology, Irbid 22110, Jordan; salazzam@just.edu.jo; 5 Department of Basic Pathological Sciences, Faculty of Medicine, Yarmouk University, Irbid 21163, Jordan; reema.karasneh@yu.edu.jo

**Keywords:** COVID-19, obesity, body mass index, mortality, hospitalized patients, secondary analysis, Jordan

## Abstract

**Highlights:**

**What are the main findings?**

- In 1399 adults hospitalized during 2020–2021, obesity (BMI ≥ 30 kg/m^2^) showed no statistically clear adjusted association with in-hospital mortality (primary adjusted OR 1.124, 95% CI 0.892–1.416).
- Secondary categorical, continuous, spline, and risk-ratio analyses were similarly imprecise; confidence intervals, particularly at BMI extremes, remained compatible with clinically meaningful differences.

**What are the implications of the main findings?**

- The findings concern prognosis conditional on hospitalization in a single early-pandemic referral cohort and should not be interpreted as evidence of equivalence or absence of an obesity effect.
- Transferability to later variant, vaccination, and treatment eras is limited, and the unresolved derivation differences between the parent cohort and current archived extract warrant cautious interpretation.

**Abstract:**

Background and Objectives: A previous analysis of the same Jordanian hospital cohort reported obesity as nonsignificant in a broad mortality model but did not characterize BMI dose–response or nonlinearity. We examined BMI and in-hospital mortality in a focused secondary analysis of adults hospitalized during 2020–2021. Materials and Methods: The archived extract contained 1399 adults and 559 deaths. The primary exposure was obesity (BMI ≥ 30 vs. <30 kg/m^2^). Logistic regression adjusted for age using a four-knot restricted cubic spline, sex, and smoking status; an expanded model additionally included comorbidities. Modified Poisson regression estimated adjusted risk ratios. Results: Mortality was 41.4% with obesity and 38.8% without obesity. The primary adjusted OR was 1.124 (95% CI 0.892–1.416; *p* = 0.322) and adjusted RR was 1.066 (0.938–1.212; *p* = 0.327). BMI-category and spline global tests were nonsignificant, but intervals were wide at the extremes. Conclusions: No statistically clear BMI–mortality association was identified in this selected early-pandemic hospitalized cohort. The findings do not establish equivalence and may not transfer to later pandemic eras; interpretation is limited by unresolved derivation differences from the parent cohort and unavailable acute-severity and timing variables.

## 1. Introduction

Coronavirus disease 2019 (COVID-19) caused substantial global morbidity and mortality and placed major pressure on health systems worldwide [1]. Body mass index (BMI) is a simple anthropometric measure used to classify obesity, but it does not directly measure body composition or fat distribution [2]. Obesity is associated with multiple cardiometabolic comorbidities [3] and with inflammatory and immune alterations that are biologically relevant to severe infection [4,5]. SARS-CoV-2 pathogenesis is multisystemic, and the relationship between adiposity and outcome may therefore differ according to the clinical stage and population being studied [6,7].

Evidence must be interpreted according to the underlying target population. Population studies address risks of infection, hospitalization, or death after infection, whereas hospital cohorts estimate prognosis conditional on admission. ICU cohorts and extracorporeal membrane oxygenation (ECMO) cohorts represent still more selected clinical populations. Early hospital and ICU studies linked higher BMI with severe respiratory support requirements [8,9], while an ECMO-selected cohort reported no mortality disadvantage associated with obesity [10]. Other hospitalized cohorts reported null or apparently favorable mortality associations after adjustment [11,12]. These settings should not be treated as interchangeable.

Broader evidence generally supports increased COVID-19 risk with obesity. A 2024 meta-analysis of 199 studies reported higher mortality among patients with obesity [13], and a meta-analysis restricted to prospective studies also found increased mortality, although heterogeneity remained substantial [14]. More recent primary data show that this relationship remains setting-dependent: a 2025 national hospital cohort reported higher adjusted inpatient mortality with obesity [15], while a separate 2025 national study documented marked changes in hospitalized patient characteristics and severity across SARS-CoV-2 variant eras [16]. These later data underscore that a cohort recruited in 2020–2021 should be interpreted as historical rather than representative of all subsequent pandemic phases.

BMI also has measurement limitations. Visceral adiposity and ectopic fat may be more closely related to severe disease than BMI alone [17,18]. In addition, analyses restricted to hospitalized patients can be affected by selection or collider bias because obesity, age, comorbidity, access to care, and acute disease severity may all influence admission [19]. Collider bias is therefore a possible source of distortion in hospital-based estimates, not a demonstrated explanation for any particular result.

The King Abdullah University Hospital (KAUH) COVID-19 cohort underlying the present study has previously been used to examine broad predictors of in-hospital mortality, invasive mechanical ventilation, and acid-base disturbances [20,21,22]. Importantly, Karasneh et al. included obesity among candidate mortality risk factors and reported that obesity was not statistically significant in their final multivariable model [20]. The prior work therefore did more than describe BMI; however, obesity was considered as one binary candidate variable within a broad prognostic analysis and the study did not specifically evaluate BMI severity categories, continuous dose–response, nonlinear associations, alternative reference categories, relative-risk estimation, or age-stratified effects.

The present study asks a narrower question: among adults hospitalized with COVID-19 at KAUH during 2020–2021, was obesity (BMI ≥ 30 versus <30 kg/m^2^) associated with in-hospital death after adjustment for core demographic and smoking covariates? Binary obesity was the primary exposure. BMI categories, continuous BMI, restricted cubic splines, alternative category references, and subgroup/sensitivity analyses were secondary or exploratory assessments intended to characterize the precision and shape of the association rather than to establish a new causal effect.

## 2. Materials and Methods

### 2.1. Study Design, Data Source, and Relationship to Previous Publications

This single-center retrospective secondary analysis used an archived de-identified analytic extract derived from the COVID-19 cohort established at KAUH, a tertiary referral hospital in northern Jordan. The parent cohort covered admissions from 20 September 2020 through 8 August 2021 and has been described previously [20,21,22]. The present study reuses part of that cohort for a BMI-focused question and should not be interpreted as an independent patient cohort.

### 2.2. Participants and Analytic Sample

**Figure 1:**
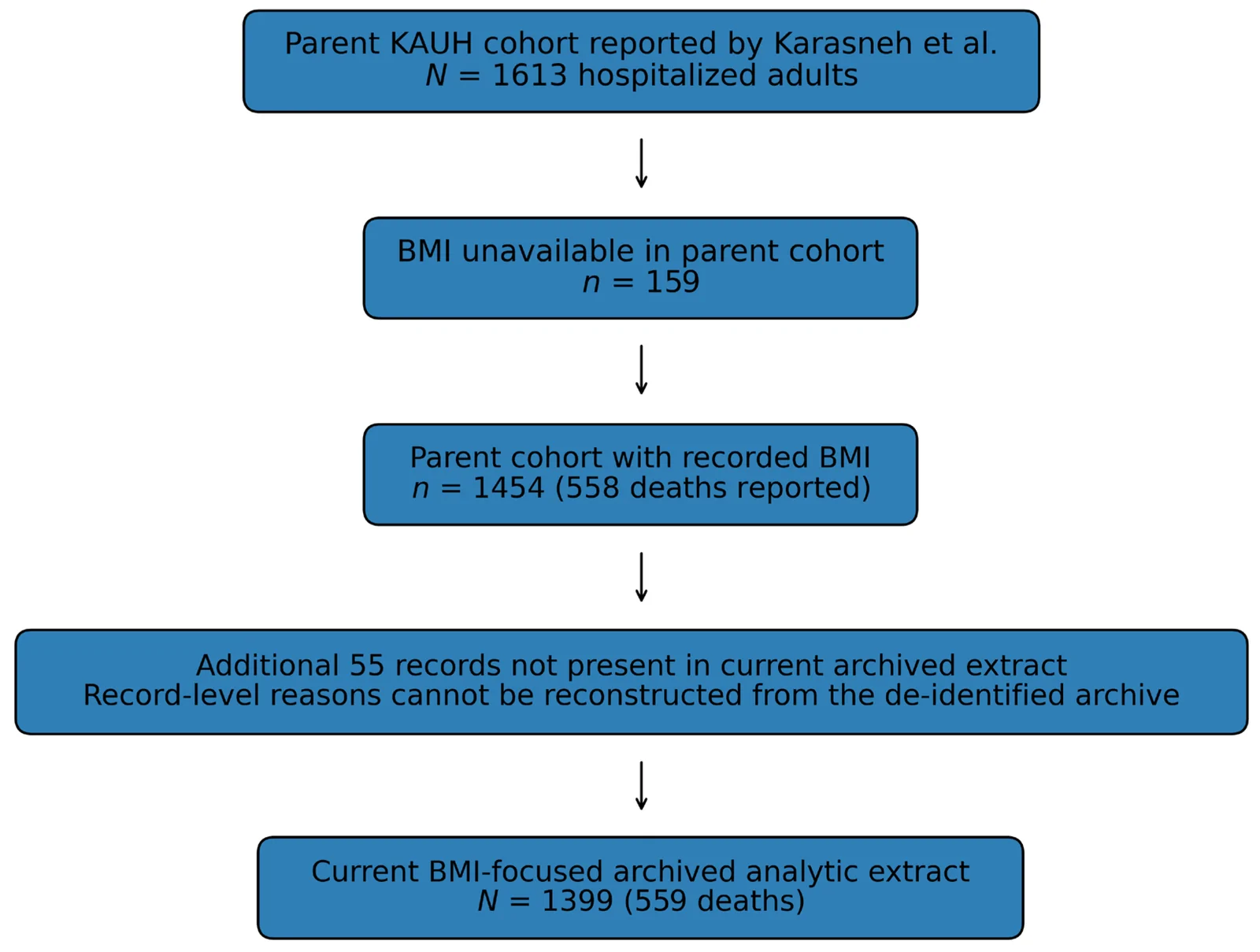
Available-data derivation of the current archived analytic extract. The figure describes dataset provenance rather than a verified exclusion flow. The record-level reasons for the additional 55-record difference and the one-death discrepancy cannot be reconstructed from the de-identified archive [20].

The parent cohort included adults aged 18 years or older with PCR-confirmed SARS-CoV-2 infection; patients younger than 18 years and those with asymptomatic or mild illness were excluded as previously described [20]. Karasneh et al. reported 1613 eligible hospitalized adults, 609 deaths, and 159 patients with unknown BMI. Thus, 1454 patients had recorded BMI and 558 deaths occurred among those BMI-recorded patients [20]. The archived extract available for the present analysis contains 1399 records and 559 deaths. The parent publication and current archive therefore cannot be reconciled by simple exclusion of missing-BMI records: for example, the parent report contained 227 normal-weight patients with 93 deaths, whereas the current archive contains 213 normal-weight patients with 94 deaths. The reason for the residual 55-record difference and the one-death discrepancy cannot be reconstructed from the current de-identified archive because patient identifiers, version history, and the original screening/exclusion log are not retained. Figure 1 summarizes the available-data derivation and explicitly distinguishes documented counts from the unresolved version difference.

Because the omitted 55 records are unavailable, their characteristics cannot be compared with the included records. If their absence was jointly related to BMI and mortality, selection bias could shift the association in either direction; its magnitude and direction cannot be quantified from the available data. The archive also does not contain patient identifiers, so unique-patient status and repeat admissions cannot be independently verified. A single binary final-disposition field is present for every archived record, but there is no transfer-status variable with which to independently verify final disposition for transfers. The study sample size was therefore determined by the fixed archived extract; no retrospective power calculation was performed.

### 2.3. BMI Ascertainment and Exposure Definitions

The parent cohort publication states that height and weight were extracted from the electronic hospital record and BMI was calculated as weight (kg)/height^2^ (m^2^) [20]. The current archived extract retains calculated BMI but not the separate height/weight fields, measurement timestamps, or metadata indicating whether measurements were directly measured, patient-reported, or clinically estimated. Consequently, those aspects of anthropometric ascertainment cannot be independently reconstructed. All 1399 records contained a numeric BMI. The observed range was 15.43–60.98 kg/m^2^; inspection identified no nonnumeric or physiologically implausible values requiring exclusion.

The primary exposure compared obesity (BMI ≥ 30 kg/m^2^) with BMI < 30 kg/m^2^. Secondary categories were defined using nonoverlapping intervals: underweight, BMI < 18.5 kg/m^2^; normal weight, 18.5 ≤ BMI < 25 kg/m^2^; overweight, 25 ≤ BMI < 30 kg/m^2^; obesity class I, 30 ≤ BMI < 35 kg/m^2^; obesity class II, 35 ≤ BMI < 40 kg/m^2^; and obesity class III, BMI ≥ 40 kg/m^2^ [2]. Only four patients were underweight, so they were retained in the primary binary analysis but excluded from the category-based regression, where a separate underweight coefficient would be unstable. The categorical model used normal weight as the primary reference and overweight as an alternative reference sensitivity analysis. BMI was also modeled continuously per 5 kg/m^2^ and with a four-knot restricted cubic spline [23].

### 2.4. Outcome

The sole outcome was in-hospital death. In the archived variable Discharge_dead, 1 denotes discharge alive/survival and 0 denotes death. For every logistic and modified Poisson analysis, we created death = 1 − Discharge_dead, so that death = 1 and survival = 0. This recoding yielded 559 deaths and 840 survivors. ICU admission, length of stay, admission date, time to death/discharge, and early-death timing were not available in the archived extract; therefore, time-to-event analyses and sensitivity analyses excluding deaths within 24–48 h could not be performed.

### 2.5. Covariates

The parent cohort reports that comorbidities were identified from related International Classification of Diseases codes and that clinical variables were abstracted from electronic records [20]. The present archive contains age, sex, smoking status, hypertension, diabetes mellitus, dyslipidemia, ischemic heart disease, atrial fibrillation, heart failure, cerebrovascular accident, chronic kidney disease (CKD), malignancy, asthma, chronic obstructive pulmonary disease (COPD), chronic liver disease, immunocompromised status, and presenting symptoms. Cardiovascular disease was defined for this secondary analysis as any history of ischemic heart disease, atrial fibrillation, heart failure, or cerebrovascular accident. Smoking codes were interpreted using the parent publication: 0 = nonsmoker, 1 = current smoker, and 2 = ex-smoker [20]. All three levels were retained in adjusted models with nonsmoker as the reference. The archived extract does not preserve the source-validation rules used for individual symptom fields.

### 2.6. Statistical Analysis

Continuous variables were summarized with medians and interquartile ranges (IQRs) and categorical variables with counts and percentages. Baseline comparisons used the Mann–Whitney U test for continuous variables and Pearson chi-square tests without continuity correction for categorical variables.

The target estimand was a conditional adjusted association between BMI and in-hospital death among already-hospitalized adults, not a causal total or direct effect of obesity. The primary adjusted logistic model included obesity, sex, smoking status (nonsmoker/current/ex-smoker), and age modeled flexibly. A likelihood-ratio comparison of a linear age term with a restricted cubic spline provided evidence against strict linearity (LR χ^2^ = 6.36, 2 df; *p* = 0.042); therefore, age was modeled with a four-knot restricted cubic spline (3 spline degrees of freedom), with boundary knots at the 5th and 95th percentiles and interior knots at the 35th and 65th percentiles (33, 57, 70, and 84 years). The primary model can be written as logit[P(death = 1)] = β0 + β1(obesity) + s(age) + β2(female) + β3(current smoker) + β4(ex-smoker), with male sex, nonsmoking, and BMI < 30 kg/m^2^ as reference levels. An expanded conditional model additionally adjusted for hypertension, diabetes, cardiovascular disease, CKD, malignancy, and dyslipidemia. Because these conditions may be downstream of long-term adiposity, differences between the primary and expanded models are interpreted as specification differences rather than total-versus-direct causal effects.

Because mortality was common, a modified Poisson model with log link and HC0 robust sandwich variance estimated adjusted risk ratios using the primary covariate set [24]. A broader-comorbidity sensitivity model additionally included asthma, COPD, chronic liver disease, and immunocompromised status. Secondary BMI analyses used the primary adjustment set. The BMI restricted cubic spline used four knots (3 spline degrees of freedom), with boundary knots at the 5th and 95th percentiles and interior knots at the 35th and 65th percentiles (22.58, 27.68, 31.71, and 41.62 kg/m^2^). Likelihood-ratio tests assessed the global BMI contribution and departure from linearity. Interaction analyses evaluated obesity × age-spline and obesity × sex terms. Age-stratified models (<65 and ≥65 years) adjusted for continuous age within stratum, sex, and smoking status. Influence was assessed using Cook’s distance and DFBETAs, with an additional analysis excluding the highest 1% of Cook’s-distance observations. An E-value was calculated for the primary adjusted risk ratio as a descriptive sensitivity analysis for unmeasured confounding [25]. Quantitative bias analysis for collider selection was not parameterized because the archive contains only hospitalized patients and lacks the nonhospitalized selection data required to specify a defensible selection model. All models were checked for convergence. Analyses used the available sample for each model: *N* = 1399 for the binary/continuous/spline models and *N* = 1395 (557 deaths) for category models excluding four underweight observations. Analyses were conducted in Python 3.13 using statsmodels 0.14.6 and patsy 1.0.2. Analysis code and a variable data dictionary are provided as Supplementary Materials.

### 2.7. Ethical Approval and Reporting

The underlying KAUH COVID-19 cohort was reported as having received approval from the Institutional Review Board of Jordan University of Science and Technology, Irbid, Jordan, on 30 December 2021 (approval number 27/137/2021) [20,21,22]. The present study is a retrospective secondary analysis of an archived de-identified analytic extract derived from that cohort. The Institutional Review Board determined that secondary analysis of the de-identified dataset did not require additional informed consent. No new participant contact, study-specific intervention, or additional medical-record extraction was undertaken for this analysis. Reporting follows the STROBE recommendations for observational studies [26].

## 3. Results

### 3.1. Cohort Characteristics

The archived extract contained 1399 records, including 643 (46.0%) with BMI ≥ 30 kg/m^2^ and 559 deaths (40.0%). Mortality was 266/643 (41.4%) among patients with obesity and 293/756 (38.8%) among those with BMI < 30 kg/m^2^ (*p* = 0.320). Median age was 64 years in both groups, but the obesity group contained fewer men and current smokers and more hypertension, diabetes, cardiovascular disease, and asthma. Median BMI was 26.57 kg/m^2^ (IQR 24.69–28.06) in the BMI < 30 group and 34.11 kg/m^2^ (31.83–37.11) in the obesity group. All variables shown in Table 1 were complete (missing n = 0).

### 3.2. Primary and Expanded Binary Obesity Analyses

The crude obesity–mortality association was OR 1.115 (95% CI 0.900–1.382; *p* = 0.320). After flexible adjustment for age, sex, and three-level smoking status, the primary adjusted OR was 1.124 (0.892–1.416; *p* = 0.322). The expanded conditional model produced OR 1.134 (0.894–1.438; *p* = 0.301). Modified Poisson regression with the primary adjustment set yielded RR 1.066 (0.938–1.212; *p* = 0.327). Thus, the point estimates were consistently above one but imprecise and compatible with both little association and clinically meaningful harm. Table 2 reports sample sizes and event counts for each model.

### 3.3. Expanded Conditional Model

Table 3 presents the expanded conditional model. Age was associated with mortality overall (global spline LR χ^2^ = 84.10, 3 df; *p* < 0.001), but no single age OR is reported because age was modeled nonlinearly. Current smoking, CKD, and malignancy had positive adjusted associations with mortality in this specification. These covariate coefficients are conditional associations within the model and are not presented as independently validated predictors.

### 3.4. BMI Categories, Continuous BMI, and Nonlinearity

The category-based model excluded four underweight patients (two deaths), leaving *N* = 1395 and 557 deaths. With normal weight as the reference, none of the higher BMI categories had a statistically clear association with mortality; the global category test was LR χ^2^ = 3.48 (4 df; *p* = 0.481). Because normal weight had the highest crude mortality among the adequately sized categories, an alternative-reference sensitivity analysis used overweight as the reference. Results remained imprecise: for example, class III obesity versus overweight had OR 1.305 (95% CI 0.799–2.131). Table 4 shows both reference parameterizations.

**Figure 2:**
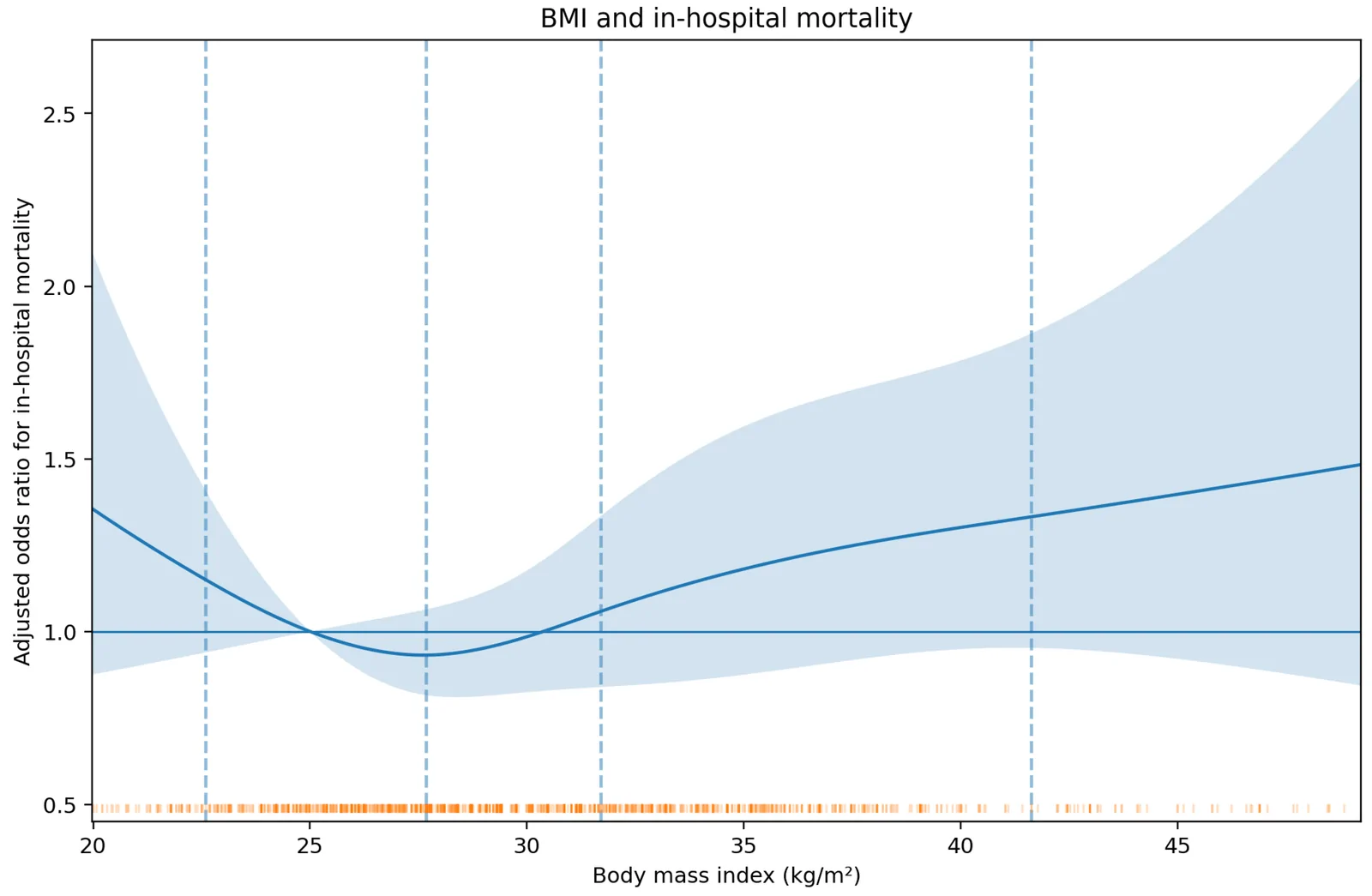
Restricted cubic spline association between BMI and in-hospital mortality, adjusted for age spline, sex, and smoking status. The curve shows adjusted odds ratios relative to BMI 25 kg/m^2^ with pointwise 95% confidence intervals. Dashed vertical lines mark the four BMI knots (22.58, 27.68, 31.71, and 41.62 kg/m^2^). Rug marks show observed BMI values. For display, the *x*-axis is restricted to the 1st–99th percentiles (19.97–49.23 kg/m^2^); all observations were included in model fitting. Confidence intervals widen at the sparsely populated extremes.

When BMI was modeled continuously, the adjusted OR per 5 kg/m^2^ was 1.068 (95% CI 0.968–1.178; *p* = 0.192). The BMI restricted cubic spline showed no statistically clear global association (LR χ^2^ = 4.69, 3 df; *p* = 0.196) and no statistically clear departure from linearity (LR χ^2^ = 2.99, 2 df; *p* = 0.224). Figure 2 displays pointwise 95% confidence intervals and the observed BMI distribution. The widening interval at the tails reflects sparse data and does not establish a dose–response pattern.

### 3.5. Age, Sex, and Additional Sensitivity Analyses

Age-stratified estimates were similar but imprecise: among patients < 65 years (*N* = 734; 208 deaths), the obesity OR was 1.085 (95% CI 0.775–1.518; *p* = 0.635); among patients ≥ 65 years (*N* = 665; 351 deaths), the OR was 1.140 (0.829–1.567; *p* = 0.421). The age-group interaction *p* value was 0.962. A global obesity × age-spline interaction test was also nonsignificant (LR χ^2^ = 3.17, 3 df; *p* = 0.367), as was obesity × sex (LR χ^2^ = 0.60, 1 df; *p* = 0.437). These tests do not exclude clinically relevant effect modification because the subgroup intervals remain broad.

All fitted models converged. No observation had Cook’s distance > 1 (maximum 0.010); the maximum absolute DFBETA for the obesity coefficient was 0.076. Excluding the highest 1% of Cook’s-distance observations yielded OR 1.102 (95% CI 0.871–1.393; *p* = 0.419). The E-value for the primary adjusted RR was 1.33; because the confidence interval included 1, the confidence-limit E-value was 1.00. This indicates that the near-null estimate is not robust evidence against unmeasured confounding. Early-death and calendar-time sensitivities could not be performed because the archive lacks event times and admission dates.

## 4. Discussion

In this single-center secondary analysis of 1399 adults hospitalized with COVID-19 in northern Jordan during 2020–2021, the primary adjusted obesity estimate was OR 1.124 (95% CI 0.892–1.416) and the corresponding modified Poisson RR was 1.066 (0.938–1.212). Point estimates were above 1, but the intervals crossed the null and remained compatible with modest harm. Secondary category, continuous, and spline analyses did not identify a statistically clear association, but they shared the same sampling and measurement limitations and should not be viewed as independent confirmations of no effect.

The contribution of this study is incremental and analytic rather than cohort-based. Karasneh et al. had already reported that obesity was not statistically significant in the final broad mortality model from the parent KAUH cohort [20]. The present analysis extends that observation by treating BMI as the exposure of interest, using severity classes, continuous BMI, nonlinear splines, a directly interpretable risk-ratio model, an alternative overweight reference group, age-stratified analyses, and formal diagnostics. These additions refine the shape and precision of the association; they do not create a new patient cohort or overturn the earlier finding.

The 40% mortality in the current extract is high and reflects the case mix and historical setting. By the study period, KAUH hospitalization criteria had shifted toward moderate, severe, and critical illness, and the hospital served as a major tertiary referral center in northern Jordan [20]. The cohort also spans a period before later Omicron-era disease patterns and during changing treatment and vaccination availability. National data have shown substantial changes in hospitalized case mix and severity across variant eras [16], and a 2025 hospital cohort found an adverse adjusted obesity–mortality association in a later setting [15]. Accordingly, the current estimate should not be extrapolated to contemporary hospitalized populations.

Selection into hospitalization remains a central interpretive issue. Conditioning on admission can distort exposure–outcome associations when obesity and other determinants of prognosis also affect hospitalization [19]. This is a plausible source of bias rather than a proven explanation of the observed estimate, and the current archive does not contain the nonhospitalized population needed for quantitative selection-bias modeling. The unexplained reduction from 1454 BMI-recorded patients in the parent publication to 1399 records in the current archive introduces an additional selection concern. Because excluded records are unavailable, neither the direction nor magnitude of that bias can be estimated.

The crude normal-weight mortality proportion (44.1%) exceeded that in the overweight and obesity classes. This could reflect chance, age/comorbidity structure, frailty, illness-related weight loss, or other forms of reverse causation. The archive does not contain pre-illness weight trajectories, nutritional status, or direct frailty measures. Re-estimating the categorical model with overweight as the reference did not produce a statistically clear association, but the class III obesity interval remained wide (OR 1.305, 95% CI 0.799–2.131), allowing substantial differences in either direction. Similarly, nonsignificant spline and interaction tests do not exclude nonlinear effects or effect modification.

The primary and expanded models produced similar obesity point estimates, but that numerical similarity does not rule out overadjustment, residual confounding, or measurement error. Hypertension, diabetes, cardiovascular disease, CKD, and dyslipidemia may partly lie on pathways linking long-term adiposity with outcome, so the expanded model is best interpreted as a more heavily conditioned association rather than a direct causal effect. Conversely, acute severity, vaccination, calendar time, treatment context, and socioeconomic factors were unavailable and could not be adjusted for. Dyspnea or fever alone would not be adequate substitutes for objective baseline severity, so they were not used to claim control of acute severity in the revised primary model.

BMI itself is also an imperfect exposure. The parent cohort calculated BMI from height and weight recorded in the EHR [20], but the current archive does not retain the measurement method or timing, and bed-bound or critically ill patients may have less reliable anthropometry. Visceral adiposity may be more informative than BMI [17,18]. Selected ICU studies have reported differing associations, including spline-based analyses consistent with an obesity-paradox pattern [27], while population data have described J-shaped associations and stronger BMI effects in younger adults [28]. These differences further support careful separation of population risk, hospital prognosis, and highly selected ICU/ECMO outcomes.

### Strengths and Limitations

Strengths include 559 mortality events, complete BMI and modeled covariates within the available extract, explicit reuse of the parent cohort, flexible adjustment for nonlinear age, risk-ratio estimation for a common outcome, category and spline analyses, an alternative reference group, age-stratified analyses, influence diagnostics, and provision of analysis code and a variable data dictionary. The revised analysis also avoids time-to-event methods because event times are not available.

Several limitations materially constrain interpretation. First, this is a retrospective single-center analysis from a tertiary referral hospital in northern Jordan and is not nationally representative. Second, the current archived extract cannot be fully reconciled with the parent publication: 55 additional BMI-recorded records are absent and the death count differs by one, while patient-level version history and exclusion reasons are unavailable. The resulting selection bias cannot be quantified. Third, unique patients versus repeat admissions and transfer disposition cannot be independently verified because identifiers and a transfer flag are absent. Fourth, the archive lacks admission date, vaccination status, viral variant, objective oxygenation/respiratory-rate measures, treatment timing, time to death/discharge, and early-death timing; thus calendar-era, acute-severity, early-death, and time-to-event analyses are not possible. Fifth, the archive does not retain height/weight measurement timing or measured-versus-estimated status. Sixth, several comorbidities are sparsely recorded and dyslipidemia is unusually uncommon, raising concern for under-ascertainment. Seventh, all secondary models reuse the same extract and therefore share its selection, measurement, and residual-confounding limitations. Finally, the observational design and hospital conditioning preclude causal interpretation.

## 5. Conclusions

Among adults hospitalized at a tertiary referral hospital in northern Jordan during 2020–2021, obesity was not associated with a statistically clear difference in in-hospital mortality after core adjustment (OR 1.124, 95% CI 0.892–1.416; adjusted RR 1.066, 95% CI 0.938–1.212). The confidence intervals remain compatible with modest harm, and wider category-specific intervals permit larger differences at BMI extremes. These historical hospital-based results should not be interpreted as equivalence, absence of an obesity effect, or evidence about present-day predictive usefulness. Their transferability to later variant, vaccination, and treatment eras is limited, and unresolved derivation differences from the parent cohort warrant additional caution.

## Figures and Tables

**Table 1:** Baseline characteristics according to obesity status.

Characteristic	BMI < 30 kg/m^2^ (n = 756)	BMI ≥ 30 kg/m^2^ (n = 643)	*p* Value
Age, years, median (IQR)	64 (50.75–73.00)	64 (54.00–74.00)	0.136
BMI, kg/m^2^, median (IQR)	26.57 (24.69–28.06)	34.11 (31.83–37.11)	—
Male sex, n (%)	506 (66.9)	314 (48.8)	<0.001
Smoking status, n (%)			0.011
Nonsmoker	539 (71.3)	494 (76.8)	
Current smoker	124 (16.4)	70 (10.9)	
Ex-smoker	93 (12.3)	79 (12.3)	
Hypertension, n (%)	392 (51.9)	440 (68.4)	<0.001
Diabetes mellitus, n (%)	347 (45.9)	354 (55.1)	<0.001
Cardiovascular disease, n (%)	193 (25.5)	203 (31.6)	0.012
Chronic kidney disease, n (%)	55 (7.3)	62 (9.6)	0.111
Malignancy, n (%)	69 (9.1)	44 (6.8)	0.118
Dyslipidemia, n (%)	32 (4.2)	42 (6.5)	0.056
Asthma, n (%)	15 (2.0)	30 (4.7)	0.005
COPD, n (%)	10 (1.3)	5 (0.8)	0.324
Chronic liver disease, n (%)	2 (0.3)	4 (0.6)	0.308
Immunocompromised, n (%)	43 (5.7)	22 (3.4)	0.045
Cough, n (%)	555 (73.4)	470 (73.1)	0.894
Dyspnea, n (%)	560 (74.1)	482 (75.0)	0.704
Fever, n (%)	465 (61.5)	395 (61.4)	0.976
Diarrhea, n (%)	98 (13.0)	97 (15.1)	0.253
In-hospital death, n (%)	293 (38.8)	266 (41.4)	0.320

Note: Cardiovascular disease was defined as a history of ischemic heart disease, atrial fibrillation, heart failure, or cerebrovascular accident. COPD, chronic obstructive pulmonary disease; IQR, interquartile range. All displayed variables had valid n equal to the column total and missing n = 0.

**Table 2:** Binary obesity association with in-hospital mortality across primary and sensitivity models.

Analysis	*N*	Deaths	Effect Estimate (95% CI)	*p* Value
Unadjusted logistic	1399	559	OR 1.115 (0.900–1.382)	0.320
Primary adjusted logistic	1399	559	OR 1.124 (0.892–1.416)	0.322
Expanded conditional logistic	1399	559	OR 1.134 (0.894–1.438)	0.301
Primary modified Poisson	1399	559	RR 1.066 (0.938–1.212)	0.327
Expanded modified Poisson	1399	559	RR 1.059 (0.931–1.204)	0.386
Broader-comorbidity sensitivity	1399	559	OR 1.143 (0.900–1.452)	0.272

Note: Primary adjustment = age modeled with a four-knot restricted cubic spline, sex, and smoking status (nonsmoker/current/ex-smoker). Expanded conditional adjustment additionally included hypertension, diabetes, cardiovascular disease, CKD, malignancy, and dyslipidemia. Broader-comorbidity sensitivity additionally included asthma, COPD, chronic liver disease, and immunocompromised status. Modified Poisson models used HC0 robust sandwich variance.

**Table 3:** Expanded conditional logistic model for in-hospital mortality.

Variable/Reference	Adjusted OR (95% CI)	*p* Value
Obesity: BMI ≥ 30 vs. <30 kg/m^2^	1.134 (0.894–1.438)	0.301
Age, four-knot restricted cubic spline	—	<0.001
Female vs. male	0.996 (0.769–1.289)	0.974
Current smoker vs. nonsmoker	1.752 (1.241–2.475)	0.001
Ex-smoker vs. nonsmoker	1.223 (0.852–1.757)	0.275
Hypertension: yes vs. no	0.859 (0.650–1.136)	0.286
Diabetes: yes vs. no	1.087 (0.847–1.395)	0.514
Cardiovascular disease: yes vs. no	1.250 (0.948–1.649)	0.114
Chronic kidney disease: yes vs. no	2.813 (1.819–4.350)	<0.001
Malignancy: yes vs. no	2.190 (1.447–3.315)	<0.001
Dyslipidemia: yes vs. no	0.931 (0.555–1.560)	0.785

Note: *N* = 1399; deaths = 559. Age was modeled with restricted cubic splines and tested globally. ORs for comorbidities should not be interpreted as causal effects or as externally validated predictors.

**Table 4:** Mortality by BMI category and adjusted categorical associations using two reference groups.

BMI Category	*N*	Deaths, n (%)	Adjusted OR vs. Normal	Adjusted OR vs. Overweight
18.5 ≤ BMI < 25 kg/m^2^	213	94 (44.1)	Reference	1.260 (0.896–1.771)
25 ≤ BMI < 30 kg/m^2^	539	197 (36.5)	0.794 (0.565–1.116)	Reference
30 ≤ BMI < 35 kg/m^2^	379	154 (40.6)	0.901 (0.628–1.292)	1.135 (0.855–1.507)
35 ≤ BMI < 40 kg/m^2^	178	76 (42.7)	1.043 (0.676–1.609)	1.314 (0.909–1.899)
BMI ≥ 40 kg/m^2^	86	36 (41.9)	1.036 (0.602–1.783)	1.305 (0.799–2.131)

Note: *N* = 1395; deaths = 557. The model adjusted for age spline, sex, and smoking status. Four underweight patients were excluded because a separate coefficient would be unstable. Global BMI-category LR χ^2^ = 3.48, 4 df; *p* = 0.481. Wide confidence intervals, especially for class III obesity, should not be interpreted as evidence of equivalence.

## Data Availability

Patient-level data are not publicly released because of institutional, ethical, and privacy restrictions. Requests for controlled access may be directed to King Abdullah University Hospital and remain subject to institutional approval. Analysis code and a data dictionary for variables used in this secondary analysis are provided in the Supplementary Materials.

## Supplementary Materials

The following supporting information can be downloaded at https://www.mdpi.com/article/10.3390/healthcare14213739/s1, Table S1: compares the published parent cohort with the current archived extract; Table S2: provides a data dictionary for variables used in the revised analyses; Table S3: reports additional sensitivity analyses and diagnostics. Reproducible analysis code is provided as Supplementary_Analysis_Code_Healthcare_4641937.py.

## Abbreviations

BMI, body mass index; CI, confidence interval; CKD, chronic kidney disease; COPD, chronic obstructive pulmonary disease; COVID-19, coronavirus disease 2019; ECMO, extracorporeal membrane oxygenation; EHR, electronic health record; IQR, interquartile range; KAUH, King Abdullah University Hospital; OR, odds ratio; RCS, restricted cubic spline; RR, risk ratio; SARS-CoV-2, severe acute respiratory syndrome coronavirus 2; STROBE, Strengthening the Reporting of Observational Studies in Epidemiology.

## References

[1] World Health Organization WHO COVID-19 Dashboard. https://data.who.int/dashboards/covid19/.

[2] Purnell J.Q., Feingold K.R., Adler R.A., Ahmed S.F., Anawalt B., Blackman M.R., Chrousos G., Corpas E., de Herder W.W., Dhatariya K., Dungan K. (2000). Definitions, Classification, and Epidemiology of Obesity. Endotext.

[3] Yuen M.M.A. (2023). Health Complications of Obesity: 224 Obesity-Associated Comorbidities from a Mechanistic Perspective. Gastroenterol. Clin. N. Am..

[4] Ouchi N., Parker J.L., Lugus J.J., Walsh K. (2011). Adipokines in inflammation and metabolic disease. Nat. Rev. Immunol..

[5] Mehta P., McAuley D.F., Brown M., Sanchez E., Tattersall R.S., Manson J.J. (2020). COVID-19: Consider cytokine storm syndromes and immunosuppression. Lancet.

[6] Cevik M., Kuppalli K., Kindrachuk J., Peiris M. (2020). Virology, transmission, and pathogenesis of SARS-CoV-2. BMJ.

[7] Murphy N.F., MacIntyre K., Stewart S., Hart C.L., Hole D., McMurray J.J.V. (2006). Long-term cardiovascular consequences of obesity: 20-year follow-up of more than 15,000 middle-aged men and women (the Renfrew–Paisley study). Eur. Heart J..

[8] Parikh R., Garcia M.A., Rajendran I., Johnson S., Mesfin N., Weinberg J., Reardon C.C. (2020). ICU outcomes in COVID-19 patients with obesity. Ther. Adv. Respir. Dis..

[9] Simonnet A., Chetboun M., Poissy J., Raverdy V., Noulette J., Duhamel A., Labreuche J., Mathieu D., Pattou F., Jourdain M. (2020). High Prevalence of Obesity in Severe Acute Respiratory Syndrome Coronavirus-2 (SARS-CoV-2) Requiring Invasive Mechanical Ventilation. Obesity.

[10] Prasad N.R., Elkholey K., Patel N.R., Junqueira E., Cohen E.S., Whitmore S.P. (2024). Obesity associated with improved mortality of extracorporeal membrane oxygenation for severe COVID-19 pneumonia. Perfusion.

[11] Biscarini S., Colaneri M., Ludovisi S., Seminari E., Pieri T.C., Valsecchi P., Gallazzi I., Giusti E., Cammà C., Zuccaro V. (2020). The obesity paradox: Analysis from the SMAtteo COVID-19 REgistry (SMACORE) cohort. Nutr. Metab. Cardiovasc. Dis..

[12] Abumayyaleh M., Gil I.J.N., El-Battrawy I., Estrada V., Becerra-Muñoz V.M., Aparisi A., Fernández-Rozas I., Feltes G., Arroyo-Espliguero R., Trabattoni D. (2021). Does there exist an obesity paradox in COVID-19? Insights of the international HOPE-COVID-19-registry. Obes. Res. Clin. Pract..

[13] Haber R., Ghezzawi M., Puzantian H., Haber M., Saad S., Ghandour Y., El Bachour J., Yazbeck A., Hassanieh G., Mehdi C. (2024). Mortality risk in patients with obesity and COVID-19 infection: A systematic review and meta-analysis. Metabolism.

[14] Cho H., Park Y., Myung S.-K. (2024). Obesity and mortality in patients with COVID-19: A meta-analysis of prospective studies. Asia Pac. J. Clin. Nutr..

[15] Bonilla Y., High D., Rullan J.A., Tabba J., Shalmiyev R., Noris T., Folds A., Martinez A., Heller D., Diaz R. (2025). Body mass index and critical care outcomes in hospitalized COVID-19 patients—A national cohort study. PLoS ONE.

[16] Parajuli P., Phipps L.A.C., Sabo R., Alsaadawi R., Robinson A., French E., Sterling R.K., N3C Consortium (2025). The changing demographics and severity in hospitalized patients across COVID-19 variants: A national cohort study. J. Clin. Transl. Sci..

[17] Yang Y., Ding L., Zou X., Shen Y., Hu D., Hu X., Li Z., Kamel I.R. (2020). Visceral Adiposity and High Intramuscular Fat Deposition Independently Predict Critical Illness in Patients with SARS-CoV-2. Obesity.

[18] Petersen A., Bressem K., Albrecht J., Thieß H.-M., Vahldiek J., Hamm B., Makowski M.R., Niehues A., Niehues S.M., Adams L.C. (2020). The role of visceral adiposity in the severity of COVID-19: Highlights from a unicenter cross-sectional pilot study in Germany. Metabolism.

[19] Griffith G.J., Morris T.T., Tudball M.J., Herbert A., Mancano G., Pike L., Sharp G.C., Sterne J., Palmer T.M., Smith G.D. (2020). Collider bias undermines our understanding of COVID-19 disease risk and severity. Nat. Commun..

[20] Karasneh R.A., Khassawneh B.Y., Al-Azzam S., Al-Mistarehi A.-H., Lattyak W.J., Aldiab M., Kabbaha S., Hasan S.S., Conway B.R., Aldeyab M.A. (2022). Risk Factors Associated with Mortality in COVID-19 Hospitalized Patients: Data from the Middle East. Int. J. Clin. Pract..

[21] Kabbaha S., Al-Azzam S., Karasneh R.A., Khassawneh B.Y., Al-Mistarehi A.-H., Lattyak W.J., Aldiab M., Hasan S.S., Conway B.R., Aldeyab M.A. (2022). Predictors of invasive mechanical ventilation in hospitalized COVID-19 patients: A retrospective study from Jordan. Expert Rev. Respir. Med..

[22] Al-Azzam N., Khassawneh B., Al-Azzam S., Karasneh R.A., Aldeyab M.A. (2023). Acid-base imbalance as a risk factor for mortality among COVID-19 hospitalized patients. Biosci. Rep..

[23] Harrell F.E. (2015). Regression Modeling Strategies: With Applications to Linear Models, Logistic and Ordinal Regression, and Survival Analysis.

[24] Zou G. (2004). A modified Poisson regression approach to prospective studies with binary data. Am. J. Epidemiol..

[25] VanderWeele T.J., Ding P. (2017). Sensitivity Analysis in Observational Research: Introducing the E-Value. Ann. Intern. Med..

[26] von Elm E., Altman D.G., Egger M., Pocock S.J., Gøtzsche P.C., Vandenbroucke J.P. (2007). The Strengthening the Reporting of Observational Studies in Epidemiology (STROBE) Statement: Guidelines for reporting observational studies. PLoS Med..

[27] Perez A.V., Viana M.V., Thomazini L.D., Loss S.H., de Machado F.C.R., Nascimento A.G.D., Kropidlofscky A.P., Gerchman F., Leitão C.B., Rech T.H. (2024). BMI and mortality in critically ill patients with COVID-19: Another brick in the wall of the obesity paradox. Obesity.

[28] Kang I.S., Kong K.A. (2021). Body mass index and severity/fatality from coronavirus disease 2019: A nationwide epidemiological study in Korea. PLoS ONE.

