# Supplementary material for: Body Mass Index and Mortality in Adults Hospitalized with COVID-19 at a Tertiary Referral Hospital in Northern Jordan, 2020–2021: A Single-Center Retrospective Secondary Analysis
Source: Healthcare (Basel). 2026 Nov 3;14(21):3739. doi: 10.3390/healthcare14213739 (PMC13762495; doi:10.3390/healthcare14213739)
Supplement: Supplementary file 1 [file healthcare-14-03739-s001.zip › healthcare-4641937-supplementary.pdf]

Supplementary Materials

Body Mass Index and Mortality in Adults Hospitalized with COVID-19 at a Tertiary Referral Hospital in Northern Jordan, 2020–2021: A Single-Center Retrospective Secondary Analysis

Table S1. Cross-version comparison of the published parent cohort and current archived extract

Item	Parent cohort (Karasneh et al. 2022)	Current archived extract	Comment
Total records	1,613	1,399	Current extract is a subset/version of the parent KAUH cohort.
Total deaths	609	559	The difference cannot be reconstructed at record level from the de-identified archive.
BMI unavailable	159	0	Parent publication reported 159 records with unknown BMI.
BMI recorded	1,454	1,399	Current extract is 55 records smaller than the BMI-recorded parent subset.
Underweight (<18.5)	4; 2 deaths	4; 2 deaths	Same count in both versions.
Normal weight	227; 93 deaths	213; 94 deaths	One-death difference despite fewer records indicates that exclusions alone do not fully explain version differences.
Overweight	554; 197 deaths	539; 197 deaths	Deaths unchanged; record count differs.
Obesity (>=30)	669; 266 deaths	643; 266 deaths	Deaths unchanged; record count differs.

Note: Parent-cohort figures are taken from Karasneh et al. (2022; doi:10.1155/2022/9617319). The current archive contains no patient identifiers or original exclusion log, so the 55-record residual difference and one-death discrepancy cannot be reconciled from the files available for this revision.

Table S2. Data dictionary for variables used in the revised analyses

Archived variable	Coding/derivation used	Role in analysis	Source/metadata note
Age	Continuous years	Covariate; modeled with a 4-knot restricted cubic spline	No missing values in current extract.
Gender	1=male; 2=female	Covariate; male reference	Coding consistent with parent-cohort categories.
BMI	Continuous kg/m^2	Primary exposure and secondary categorical/continuous/spline exposure	Parent publication states height and weight were extracted from EHR and BMI calculated as kg/m^2. Current archive does not retain height, weight, timing, or measured-versus-estimated metadata.
Smoking	0=nonsmoker; 1=current smoker; 2=ex-smoker	Three-level covariate; nonsmoker reference	Clinical labels recovered from the parent-cohort publication.
Discharge_dead	1=discharged/survived; 0=died	Recoded as death=1-Discharge_dead for all logistic/Poisson models	Current extract: 559 deaths and 840 survivors.
HTN	0=no; 1=yes	Expanded-model covariate	Parent publication states comorbidities were identified

			using related ICD codes.
DM	0=no; 1=yes	Expanded-model covariate	As above.
IHD, Afib, HF, CVA	0=no; 1=yes	Combined as cardiovascular disease = any of the four	As above.
CKD	0=no; 1=yes	Expanded-model covariate	As above.
Malignancy	0=no; 1=yes	Expanded-model covariate	As above.
Dyslipid	0=no; 1=yes	Expanded-model covariate	As above; low recorded prevalence may reflect under-ascertainment.
Asthma, COPD, Liver_disease, immuncomprimissed	0=no; 1=yes	Broader-comorbidity sensitivity model	As above; several conditions are sparse in the current extract.
Cough, SOB, Fever, Diarrhea	0=absent; 1=present	Descriptive variables	Abstracted clinical presentation variables; validation metadata are not retained in current archive.

Table S3. Additional sensitivity analyses and model diagnostics

Analysis	N	Deaths	Estimate/test	Interpretation
Primary adjusted logistic model	1,399	559	OR 1.124 (0.892-1.416); p=0.322	Core adjustment: age spline, sex, smoking status.
Expanded conditional logistic model	1,399	559	OR 1.134 (0.894-1.438); p=0.301	Adds HTN, diabetes, cardiovascular disease, CKD, malignancy, dyslipidemia.
Broader-comorbidity sensitivity model	1,399	559	OR 1.143 (0.900-1.452); p=0.272	Additionally adds asthma, COPD, chronic liver disease, immunocompromised status.
Primary modified Poisson model	1,399	559	RR 1.066 (0.938-1.212); p=0.327	HC0 robust sandwich variance.
Age linearity check	1,399	559	LR chi-square=6.36, df=2, p=0.042	Evidence against a strictly linear age term; age modeled flexibly in primary analyses.
Age <65 years	734	208	OR 1.085 (0.775-1.518); p=0.635	Age-stratified sensitivity analysis.
Age >=65 years	665	351	OR 1.140 (0.829-1.567); p=0.421	Age-group interaction p=0.962.
Obesity x age-spline interaction	1,399	559	LR chi-square=3.17, df=3, p=0.367	Does not exclude clinically relevant modification because CIs remain broad.
Obesity x sex interaction	1,399	559	LR chi-square=0.60, df=1, p=0.437	No statistically clear interaction detected.
Influence sensitivity: exclude top 1% Cook's distance	1,385	548	OR 1.102 (0.871-1.393); p=0.419	Max Cook's D=0.010; no Cook's D >1.
E-value for primary adjusted RR	1,399	559	Point estimate E-value=1.33; confidence-limit E-value=1.00	Estimate is not robust to modest unmeasured confounding; CI already includes the null.
Class III obesity vs overweight reference	1,395	557	OR 1.305 (0.799-2.131)	Wide interval permits substantial harm or benefit.

Supplementary analysis code is provided separately as Supplementary_Analysis_Code_Healthcare_4641937.py. Patient-level data are not publicly released because of institutional and privacy restrictions; controlled access remains subject to KAUH approval.
